# The Tempo and Mode of Adaptation in a Complex Natural Population: the Microbiome

**DOI:** 10.1128/mSystems.00779-21

**Published:** 2021-08-24

**Authors:** Nandita Garud

**Affiliations:** a Department of Ecology and Evolutionary Biology, University of California, Los Angeles, Los Angeles, California, USA; b Department of Human Genetics, University of California, Los Angeles, Los Angeles, California, USA

## Abstract

Adaptation is a fundamental process by which populations evolve to grow more fit in their environments. Recent studies are starting to show us that commensal microbes can evolve on short timescales of days and months, suggesting that ecological changes are not the only means by which microbes in complex natural populations respond to selection pressures. However, we still lack a complete understanding of the tempo and mode of adaptation in microbiomes given the many complex forces that natural populations experience, which include ecological pressures, changes in population size, spatial structure, and fluctuations in selection pressures. Advances in modeling complex populations and scenarios will allow us to understand adaptation not only in microbiomes but also more generically in other natural populations that experience similar complexities.

## COMMENTARY

A plethora of new mutations enter the human microbiome daily (1), generating a tremendous amount of genetic variation. These genetic variants confer fundamental phenotypes not only to microbes but also to hosts, impacting the digestion of food (2), metabolism of drugs (3), and evasion of antibiotics (4). Given the functional importance of genetic variation in the microbiome, it is essential to understand the underlying population genetic processes that generate it.

Recent work is starting to show that microbiome genetic variation can change via adaptation within healthy hosts over a matter of days and months (1, 5, 6). This is exciting because it points us to the hypothesis that adaptation in the human microbiome may impact human phenotypes on short time scales and possibly is even responsible for longer-term phylosymbioses observed between microbes and their hosts (7). However, much remains to be understood about adaptation in the microbiome.

Specifically, how rapid is adaptation in the microbiome? What are the mechanisms of adaptation that influence its rate? The rates and mechanisms of adaptation are also known as tempo and mode, descriptors of evolution coined by George Gaylord Simpson in 1945 (8). The two are inextricably linked, because the mechanisms by which a population evolves can determine the rate, and vice versa. As a concrete example, HIV (9) and many other organisms with large census population sizes (10) can rapidly adapt via the rise in frequency of multiple *de novo* mutations entering the population almost simultaneously or by preexisting genetic variation that becomes beneficial in a new environment (11, 12). Similarly, in the human gut microbiome, bacteria can evolve rapidly via the daily influx of new mutations as well as by horizontal gene transfer (1, 5, 6), the latter which leverages the resources of the broader community.

To quantify the extent of adaptation in the microbiome, we need to incorporate into our models the many complexities that natural microbial communities experience. These include their ecological coexistence with hundreds of other species as well as complex forces such as fluctuating selective pressures, demographic changes, and spatial structure. Some of these features can be approximated in experimental settings, but likely not in their entirety (13). By considering more complex and realistic models, we may be able to identify adaptive events that otherwise may go undetected using statistics that rely on simpler models.

Complex forces are not unique to the microbiome. Rather, complex forces impact virtually any natural population subject to living in a community experiencing even normal seasonal changes in weather. Yet, more broadly, our understanding of adaptation in natural populations is still forming. The microbiome may in fact be one of the most exciting venues to study adaptation in a natural population because (i) microbes can evolve rapidly, (ii) we can sample replicate populations across hosts to extrapolate general principals of evolution, and (iii) we can study multiple species simultaneously. Thus, the insights made in the microbiome likely hold much relevance to our understanding of evolution in natural populations more generically.

Here, I will highlight features of the microbiome that can impact our ability to quantify the tempo and mode of adaptation. Moving forward, incorporating these features into our models will be important for quantifying adaptation in this complex natural population.

## ECOLOGICAL COMPLEXITY

While much of our knowledge about the evolutionary dynamics of microbes comes from studying one species at a time, in reality, microbial species are interconnected through a complex web of interactions in which they share metabolites and genes with one another (1, 5, 14, 15). These interactions are so important that phenotypes of a focal species can change depending on whether the species is studied in isolation or in the context of a broader community (16). Thus, the ecosystem likely modifies the evolutionary dynamics of a focal species, and vice versa. For example, new niches may form from the presence of multiple interacting players and enable rapid adaptation (17). Alternatively, community complexity may fill niches, thereby constraining rates of adaptation. To what extent the mode and tempo of adaptation of a focal microbiome species is influenced by the broader community is still an open question. New experimental and statistical techniques that can test the effects of community complexity on rates of adaptation will be needed. Additionally, temporal data in which samples are collected in dense intervals over long periods of time will be informative in identifying adaptions and diversifications of resident strains into new ecological players, as has been previously observed in the laboratory (13).

## DEMOGRAPHIC FLUCTUATIONS IN POPULATION SIZE

Natural populations experience a range of demographic forces that include bottlenecks, migration, and massive population sizes. These demographic factors can influence modalities and rates of adaptation by changing the input mutation rate and the amount of available standing genetic variation that can be leveraged for adaptation. In massive populations, for example, the combination of mutation rate and population size can result in rapid adaptation mediated by multiple beneficial mutations entering the population almost simultaneously and rising to high frequencies, also known as a soft sweep (12). In contrast, hard sweeps are associated with slower adaptation in which a single adaptive mutation rises to high frequency (12). Soft sweeps may be common in the microbiome (18), especially given its large census size (19) and abundant genetic diversity (20). Smaller populations that experience constant population sizes are more predictable and thus arguably easier to model. However, by focusing only on these more predictable scenarios, we may fail to capture the full extent of adaptation in the microbiome.

## SPATIAL STRUCTURE

Microbiomes across a wide range of environments, including oceans (21), rivers (22), soil (23), and the human gut (24), display complex spatial structure. This spatial structure has been associated with a number of critical functions, including the bioremediation of soils, carbon and nitrogen cycling, and responding to changes in diet and drugs. Despite the profound impacts of space on microbiome phenotypes, our understanding of how natural populations evolve in spatially structured environments is still nascent. More generally, while much theory has been developed on evolutionary dynamics in spatially structured populations (25), this theory remains to be fully tested in natural populations due to a general paucity of temporal and spatial data.

For example, in spatially structured environments, adaptive mutations may either be restricted to local geographic regions due to migration barriers or local niches or may spread quickly due to drift in smaller subpopulations (26). The range over which adaptive mutations can spread and the rapidity with which they arise can have implications for how quickly a population can adapt and the range of phenotypes that microbes may express across a spatial system. Thus, to fully understand adaptation in these spatially structured communities, spatially and temporally resolved data as well as statistics equipped to deal with these dimensions are needed.

## RAPID FLUCTUATIONS IN SELECTIVE PRESSURES

Natural populations experience a variety of forces that are not constant over time. For example, Darwin’s finches have experienced a rapid fluctuation in seasonal availability in food, which has resulted in an oscillation in beak size over a matter of just a few years (27). Similarly, *Drosophila* experience a periodic fluctuation in selective pressures as seasons transition from winter to summer and vice versa (28). These rapid fluctuations in selective pressures suggest that there are temporally dependent signatures of adaptation that may not be captured in a study examining fixations of adaptive alleles over long time scales.

In the human microbiome, we recently observed fixations on short time scales but found that, on longer time scales, these fixations did not seem to accrue (5). Could adaptations that appear on short time scales be specific to a given host environment and thus be purged in novel host environments?

To capture the full extent of adaptation in the microbiome, we might examine events occurring on both short and long time scales. On long time scales, we might expect sweeps to achieve fixation, whereas on short time scales, sweeps may reach only intermediate frequency due to temporal fluctuations in selective pressures (28). Denser time series data once again will be necessary to identify short-term adaptations, especially since generation times of bacteria can be as rapid as 10 generations per day (29).

## CONNECTING EXISTING MODELS WITH THE DATA

Admittedly, the complexity of natural populations makes them more difficult to model than simpler more ideal populations. To begin to model the full range of forces that impact complex populations, we can start by fitting existing models to data and identifying departures from these models. Any deviations can then subsequently fuel new modeling innovations.

**FIG 1:**
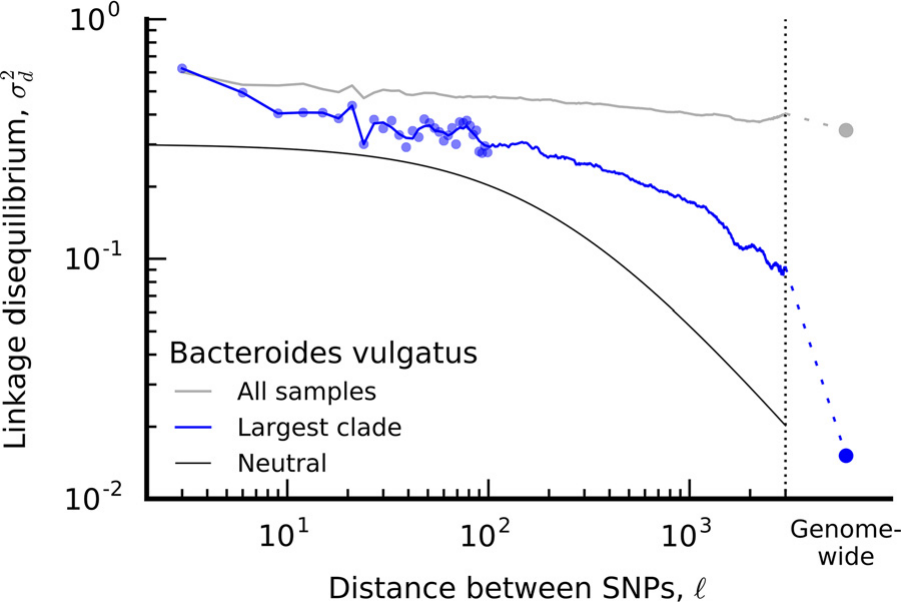
Decay in linkage disequilibrium in data versus a neutral expectation under a simple model that assumes panmixia, no selection, and a single recombination rate. The gray line “all samples” is LD measured across all lineages of Bacteroides vulgatus identified in healthy hosts analyzed previously. After correcting for clade structure in the data, the blue line is LD measured among lineages belonging to the largest clade. Despite clade correction, LD in the data continues to be elevated relative to neutral expectations. This figure is replicated from a study by Garud et al. (5).

As an example, recently, we quantified the extent of recombination in the microbiome by measuring correlations between pairs of sites by using a common population genetic statistic known as linkage disequilibrium (LD) (5). We found that LD decays over distance between pairs of single nucleotide polymorphisms (SNPs) within a gene (Fig. 1). This observation indicates that although bacteria asexually reproduce, recombination can be common. However, LD in data decays more gradually than expected under a simple null model that assumes panmixia, no selection, and a single recombination rate (30). This deviation between the data and the model suggests that additional evolutionary forces are needed to fully explain LD in the data. Could positive selection play a role? What about unaccounted population substructure or uneven recombination rates across the genome? Possibly, all of these factors and more contribute to this mismatch between theoretical predictions and patterns in the data.

To reconcile discrepancies between data and theory, simulations may be a productive way forward. By simulating, we may be able to explore a large parameter space and capture complex interactions between multiple evolutionary forces that we may not be able to model with equations or experiments in the lab. Even by simulating one species at a time and understanding each species in depth, we may start to piece together the full complexity of forces impacting the entire community. Additionally, new data, especially long-read data from which we can quantify long-range linkage, can enable us to identify additional discrepancies between the data and models. Finally, innovative statistics that enable us to quantify nontraditional signatures of adaptation will move us closer toward a comprehensive understanding of evolutionary forces shaping natural populations.

## CONCLUDING REMARKS

It is crucial to understand evolution in its natural context. If we only studied evolution in the lab, then we might never fully understand the full range of evolutionary forces acting in natural populations. However, the trade-off in studying evolution in natural populations is that because the populations are so complex, it is challenging to unravel the many interacting forces acting simultaneously. Thus, a combination of approaches is needed, as they can inform one another and will yield exciting insights into the tempo and mode of adaptation in the microbiome.

## References

[1] Zhao S, Lieberman TD, Poyet M, Kauffman KM, Gibbons SM, Groussin M, Xavier RJ, Alm EJ. 2019. Adaptive evolution within gut microbiomes of healthy people. Cell Host Microbe 25:656.e8–667.e8. doi:10.1016/j.chom.2019.03.007. 31028005 PMC6749991

[2] Hehemann J-H, Correc G, Barbeyron T, Helbert W, Czjzek M, Michel G. 2010. Transfer of carbohydrate-active enzymes from marine bacteria to Japanese gut microbiota. Nature 464:908–912. doi:10.1038/nature08937. 20376150

[3] Haiser HJ, Gootenberg DB, Chatman K, Sirasani G, Balskus EP, Turnbaugh PJ. 2013. Predicting and manipulating cardiac drug inactivation by the human gut bacterium *Eggerthella lenta*. Science 341:295–298. doi:10.1126/science.1235872. 23869020 PMC3736355

[4] Karami N, Martner A, Enne VI, Swerkersson S, Adlerberth I, Wold AE. 2007. Transfer of an ampicillin resistance gene between two *Escherichia coli* strains in the bowel microbiota of an infant treated with antibiotics. J Antimicrob Chemother 60:1142–1145. doi:10.1093/jac/dkm327. 17768176

[5] Garud NR, Good BH, Hallatschek O, Pollard KS. 2019. Evolutionary dynamics of bacteria in the gut microbiome within and across hosts. PLoS Biol 17:e3000102. doi:10.1371/journal.pbio.3000102. 30673701 PMC6361464

[6] Yaffe E, Relman D. 2019. Tracking microbial evolution in the human gut using Hi-C. Nat Microbiol 5:343–353. doi:10.1038/s41564-019-0625-0. 31873203 PMC6992475

[7] Moeller AH, Caro-Quintero A, Mjungu D, Georgiev AV, Lonsdorf EV, Muller MN, Pusey AE, Peeters M, Hahn BH, Ochman H. 2016. Cospeciation of gut microbiota with hominids. Science 353:380–382. doi:10.1126/science.aaf3951. 27463672 PMC4995445

[8] Simpson GG. 1945. Tempo and mode in evolution. Trans N Y Acad Sci 8:45–60. doi:10.1111/j.2164-0947.1945.tb00215.x. 21012247

[9] Feder AF, Rhee S-Y, Holmes SP, Shafer RW, Petrov DA, Pennings PS. 2016. More effective drugs lead to harder selective sweeps in the evolution of drug resistance in HIV-1. Elife 5:e10670. doi:10.7554/eLife.10670. 26882502 PMC4764592

[10] Messer PW, Petrov DA. 2013. Population genomics of rapid adaptation by soft selective sweeps. Trends Ecol Evol 28:659–669. doi:10.1016/j.tree.2013.08.003. 24075201 PMC3834262

[11] Pennings PS, Hermisson J. 2006. Soft sweeps II–molecular population genetics of adaptation from recurrent mutation or migration. Mol Biol Evol 23:1076–1084. doi:10.1093/molbev/msj117. 16520336

[12] Hermisson J, Pennings PS. 2005. Soft sweeps: molecular population genetics of adaptation from standing genetic variation. Genetics 169:2335–2352. doi:10.1534/genetics.104.036947. 15716498 PMC1449620

[13] Good BH, McDonald MJ, Barrick JE, Lenski RE, Desai MM. 2017. The dynamics of molecular evolution over 60,000 generations. Nature 551:45–50. doi:10.1038/nature24287. 29045390 PMC5788700

[14] D’Souza G, Shitut S, Preussger D, Yousif G, Waschina S, Kost C. 2018. Ecology and evolution of metabolic cross-feeding interactions in bacteria. Nat Prod Rep 35:455–488. doi:10.1039/c8np00009c. 29799048

[15] Groussin M, Poyet M, Sistiaga A, Kearney SM, Moniz K, Noel M, Hooker J, Gibbons SM, Segurel L, Froment A, Mohamed RS, Fezeu A, Juimo VA, Lafosse S, Tabe FE, Girard C, Iqaluk D, Nguyen LTT, Shapiro BJ, Lehtimäki J, Ruokolainen L, Kettunen PP, Vatanen T, Sigwazi S, Mabulla A, Domínguez-Rodrigo M, Nartey YA, Agyei-Nkansah A, Duah A, Awuku YA, Valles KA, Asibey SO, Afihene MY, Roberts LR, Plymoth A, Onyekwere CA, Summons RE, Xavier RJ, Alm EJ. 2021. Elevated rates of horizontal gene transfer in the industrialized human microbiome. Cell 184:205.e183–2067.e18. doi:10.1016/j.cell.2021.02.052. 33794144

[16] Datta MS, Sliwerska E, Gore J, Polz MF, Cordero OX. 2016. Microbial interactions lead to rapid micro-scale successions on model marine particles. Nat Commun 7:11965. doi:10.1038/ncomms11965. 27311813 PMC4915023

[17] Madi N, Vos M, Murall CL, Legendre P, Shapiro BJ. 2020. Does diversity beget diversity in microbiomes? Elife 9:e58999. doi:10.7554/eLife.58999. 33215610 PMC7755399

[18] Barroso-Batista J, Sousa A, Lourenço M, Bergman M-L, Sobral D, Demengeot J, Xavier KB, Gordo I. 2014. The first steps of adaptation of *Escherichia coli* to the gut are dominated by soft sweeps. PLoS Genet 10:e1004182. doi:10.1371/journal.pgen.1004182. 24603313 PMC3945185

[19] Sender R, Fuchs S, Milo R. 2016. Revised estimates for the number of human and bacteria cells in the body. PLoS Biol 14:e1002533. doi:10.1371/journal.pbio.1002533. 27541692 PMC4991899

[20] Schloissnig S, Arumugam M, Sunagawa S, Mitreva M, Tap J, Zhu A, Waller A, Mende DR, Kultima JR, Martin J, Kota K, Sunyaev SR, Weinstock GM, Bork P. 2013. Genomic variation landscape of the human gut microbiome. Nature 493:45–50. doi:10.1038/nature11711. 23222524 PMC3536929

[21] Nayfach S, Rodriguez-Mueller B, Garud N, Pollard KS. 2016. An integrated metagenomics pipeline for strain profiling reveals novel patterns of bacterial transmission and biogeography. Genome Res 26:1612–1625. doi:10.1101/gr.201863.115. 27803195 PMC5088602

[22] Doherty M, Yager PL, Moran MA, Coles VJ, Fortunato CS, Krusche AV, Medeiros PM, Payet JP, Richey JE, Satinsky BM, Sawakuchi HO, Ward ND, Crump BC. 2017. Bacterial biogeography across the Amazon River-ocean continuum. Front Microbiol 8:882. doi:10.3389/fmicb.2017.00882. 28588561 PMC5440517

[23] Crits-Christoph A, Olm MR, Diamond S, Bouma-Gregson K, Banfield JF. 2019. Soil bacterial populations are shaped by recombination and gene-specific selection across a meadow. ISME J 14:1834–1846. doi:10.1038/s41396-020-0655-x. PMC7305173 32327732

[24] Tropini C, Earle KA, Huang KC, Sonnenburg JL. 2017. The gut microbiome: connecting spatial organization to function. Cell Host Microbe 21:433–442. doi:10.1016/j.chom.2017.03.010. 28407481 PMC5576359

[25] Bradburd GS, Ralph PL. 2019. Spatial population genetics: it’s about time. Annu Rev Ecol Evol Syst 50:427–449. doi:10.1146/annurev-ecolsys-110316-022659.

[26] Zhang Q, Lambert G, Liao D, Kim H, Robin K, Tung C-k, Pourmand N, Austin RH. 2011. Acceleration of emergence of bacterial antibiotic resistance in connected microenvironments. Science 333:1764–1767. doi:10.1126/science.1208747. 21940899

[27] Grant PR, Grant BR. 2002. Unpredictable evolution in a 30-year study of Darwin’s finches. Science 296:707–711. doi:10.1126/science.1070315. 11976447

[28] Bergland AO, Behrman EL, O'Brien KR, Schmidt PS, Petrov DA. 2014. Genomic evidence of rapid and stable adaptive oscillations over seasonal time scales in *Drosophila*. PLoS Genet 10:e1004775. doi:10.1371/journal.pgen.1004775. 25375361 PMC4222749

[29] Korem T, Zeevi D, Suez J, Weinberger A, Avnit-Sagi T, Pompan-Lotan M, Matot E, Jona G, Harmelin A, Cohen N, Sirota-Madi A, Thaiss CA, Pevsner-Fischer M, Sorek R, Xavier R, Elinav E, Segal E. 2015. Growth dynamics of gut microbiota in health and disease inferred from single metagenomic samples. Science 349:1101–1106. doi:10.1126/science.aac4812. 26229116 PMC5087275

[30] Ohta T, Kimura M. 1969. Linkage disequilibrium at steady state determined by random genetic drift and recurrent mutation. Genetics 63:229–238. doi:10.1093/genetics/63.1.229. 5365295 PMC1212334

